# The maturity in fetal pigs using a multi-fluid metabolomic approach

**DOI:** 10.1038/s41598-020-76709-8

**Published:** 2020-11-16

**Authors:** Gaëlle Lefort, Rémi Servien, Hélène Quesnel, Yvon Billon, Laurianne Canario, Nathalie Iannuccelli, Cécile Canlet, Alain Paris, Nathalie Vialaneix, Laurence Liaubet

**Affiliations:** 1 INRAE, UR875 Mathématiques et Informatique Appliquées Toulouse, 31326 Castanet-Tolosan, France; 2 GenPhySE, Université de Toulouse, INRAE, ENVT, 31326 Castanet-Tolosan, France; 3 INRAE, Univ. Montpellier, LBE, 102 Avenue des étangs, 11000 Narbonne, France; 4 INTHERES, Université de Toulouse, INRAE, ENVT, Toulouse, France; 5 PEGASE, INRAE, Institut Agro, 35590 Saint Gilles, France; 6 INRAE, GENESI, 17700 Saint Pierre d’Amilly, France; 7 Axiom Platform, MetaToul-MetaboHUB, National Infrastructure for Metabolomics and Fluxomics, Toulouse, France; 8 MCAM, Muséum National d’Histoire Naturelle, CNRS, Paris, France

**Keywords:** Metabolomics, Metabolomics, Reproductive biology, Intrauterine growth

## Abstract

In mammalian species, the first days after birth are an important period for survival and the mortality rate is high before weaning. In pigs, perinatal deaths average 20% of the litter, with important economic and societal consequences. Maturity is one of the most important factors that influence piglet survival at birth. Maturity can be defined as the outcome of complex mechanisms of intra-uterine development and maturation during the last month of gestation. Here, we provide new insights into maturity obtained by studying the end of gestation at two different stages (3 weeks before term and close to term) in two breeds of pigs that strongly differ in terms of neonatal survival. We used metabolomics to characterize the phenotype, to identify biomarkers, and provide a comprehensive understanding of the metabolome of the fetuses in late gestation in three fluids (plasma, urine, and amniotic fluid). Our results show that the biological processes related to amino acid and carbohydrate metabolisms are critical for piglet maturity. We confirm the involvement of some previously described metabolites associated with delayed growth (e.g., proline and myo-inositol). Altogether, our study proposes new routes for improved characterization of piglet maturity at birth.

## Introduction

In mammalian species, the first days after birth are an important period for survival and the mortality rate is high before weaning. In humans, despite the important reduction in the mortality rate in the recent years, neonatal deaths (before 1 month after birth) still represent 47% of deaths before the age of five, i.e. about 2.5 million per year^1^. In a polytocous species like swine, this rate averages 20% of the litter in commercial lines^2^, and the most critical period for piglet survival is the perinatal period that includes birth and the first 24 h of postnatal life. Many factors have been shown to influence piglet survival at and after birth^2^. They have been related to maternal effects (e.g., intrauterine effects, farrowing duration, parity, health status), to piglet factors (e.g., genetic type, vitality at birth), to piglet characteristics that are partly under maternal control (e.g., birth weight) or to a combination of these factors. Consistently, the most important factors identified for postnatal survival are birth weight, hypothermia, the latency to first suckle, or their combinations, predisposing piglets to starvation or crushing^2,3^. Piglet maturity is also likely to be an important determinant of subsequent survival and postnatal growth^4,5^. Maturity at birth, which can be defined as complete development enabling survival at birth, is the outcome of complex intra-uterine development and maturation mechanisms that occur during the end of gestation^4^. In pigs, the maturation period is considered to be the last month of gestation (approximately 90–114 days of gestation, dg). Together with environmental conditions, physiological maturity at birth thus has major consequences for neonatal mortality highlighting the need for a deeper understanding of maturity to effectively reduce perinatal mortality.

In this context, the aim of the present study was to provide a comprehensive description of the metabolome of pig fetuses in late gestation. Metabolomics is a promising approach to investigate health and welfare in large cohorts, for phenotype characterization and for the identification on usable biomarkers: high-throughput metabolome measurements are easy to obtain and at affordable cost by $^1$H Nuclear Magnetic Resonance (NMR) and the metabolome enables the comprehensive characterization of the small molecules involved in metabolic chemical reactions. To this end, we compared the metabolomes of plasma, urine, and amniotic fluid, in 611 fetuses in two breeds of pigs, Large White (LW) and Meishan (MS), at two stages of late gestation (90 dg and 110 dg). The three fluids were chosen to represent different aspects of fetus metabolism: plasma reflects the regulation of fetal metabolism, the urine metabolome its excretory renal function, and amniotic fluid its nutritional function and mechanical protection as well as interactions with maternal and placental tissues. The two stages of gestation are representative of the maturation process in late gestation, 90 dg being the onset of fetal maturation in both breeds and 110 dg being close to term^6^. The two breeds of pigs were chosen because they strongly differ in terms of neonatal survival and can thus be used to identify differences that are possibly responsible for perinatal survival. The LW breed represents European breeds and has been genetically selected for lean growth and prolificacy. Its high rate of perinatal mortality is partly due to lower physiological maturity at birth^7^. On the contrary, the MS breed presents a low rate of mortality and is considered to be more mature at birth^8,9^. LW and MS sows were inseminated with mixed (LW and MS) semen so that pure and crossed fetuses would grow in the same uterine environment. The reciprocal crossed fetuses were expected to present an intermediate degree of maturity between LW and MS fetuses. To a lesser extent, these reciprocal crossed fetuses also allowed us to observe maternal or paternal effects or heterosis.

This study, and the search for differences between the two breeds and between the two stages of gestation, completes our previous transcriptomic and proteomic studies^10–13^ that were performed on muscle, intestinal and adipose tissues using the same experimental design. The present study also completes the blood parameters known to be associated with piglet maturity at birth (e.g., albumin and IGF-I plasma concentrations^7^). The potential and functional new biomarkers reported here can be used for genetic selection or to improve management of sow feeding in late gestation. Even if the fatty acid metabolism could not be investigated in our study (due to technical limitations regarding lipid quantification), the study allowed us to confirm some previously described metabolites associated with delayed growth and to identify important biological processes involved in piglet maturity.

## Results

A $^1$H NMR metabolomic analysis was performed on plasma, urine, and amniotic fluid collected from 611 fetuses at 90 and 110 days of gestation. Metabolic quantification was performed automatically with the R package **ASICS**. Among the 190 available metabolites in the reference library of the **ASICS** package, about 65 metabolites were identified in each fluid (i.e., 63 in plasma, 64 in urine and 68 in amniotic fluid; Supplementary Table S1; Supplementary Fig. S1). Thirty-nine metabolites were identified in all three fluids including many amino acids (e.g., glutamine, glycine, proline, and arginine) and many sugars (e.g., glucose, fructose, glucose-6-phosphate). Other metabolites were identified in only one or two fluids, e.g., leucine and isoleucine, identified only in plasma and urine, or reduced or oxidated glutathione, identified only in urine and amniotic fluid.

### Multivariate exploratory analyses

**Figure 1:**
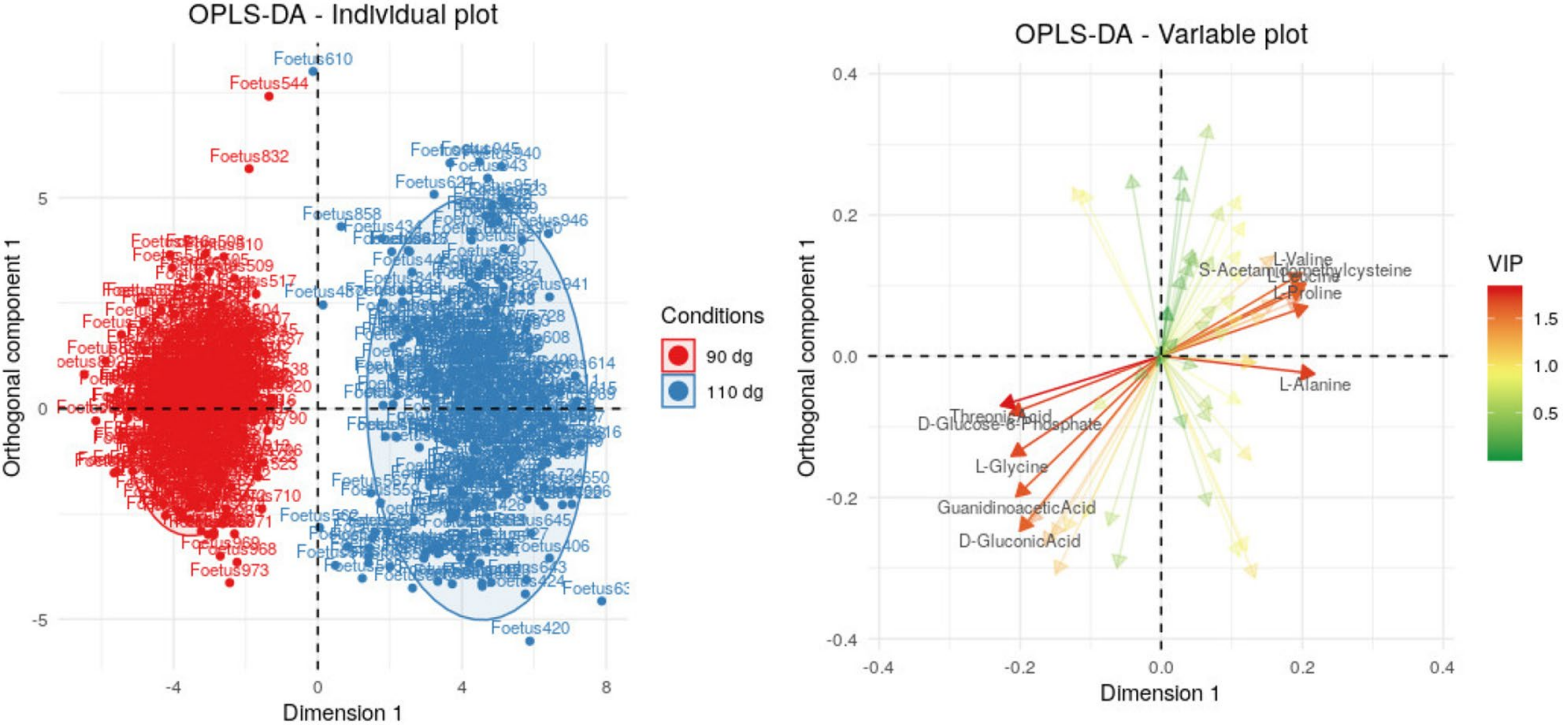
Individual and variable plots for the first two axes of the Orthogonal Projections to Latent Structures Discriminant Analyses (OPLS-DA) on $n=611$ fetuses. Figures were obtained using the quantifications from plasma spectra for both days of gestation (90 dg and 110 dg) and all genotypes (LW, MS and cross fetuses together). *VIP* Variable Influence on Projection.

Three Orthogonal Projections to Latent Structures Discriminant Analyses (OPLS-DA)^14^, one for each fluid, discriminated the two stages of gestation with good accuracy (Fig. 1; Supplementary Fig. S2), especially in the plasma where the cross-validation error was 1%. For urine and amniotic fluid, the error was slightly higher (4%) but still low, indicating a slightly less clear separation between the two groups (90 dg and 110 dg). Altogether, these results suggest that OPLS-DA can be interpreted with a high level of confidence.

Around 20 metabolites were found to be influential (Variable Influence on Projection, VIP > 1) in each fluid (23 in plasma, 21 in urine and 22 in amniotic fluid) and were consequently used for enrichment analyses of the pathways. The analyses were performed for each fluid separately, results are presented in Table 1. Only three influential metabolites were common to the three fluids (glucose-6-phosphate, fructose and guanidinoacetate; Supplementary Fig. S3). Guanidinoacetate and fructose were more concentrated at 90 dg than at 110 dg in all three fluids while glucose-6-phosphate was more concentrated at 90 dg in plasma and amniotic fluid but more concentrated at 110 dg in urine. Glucose-6-phosphate is involved in “galactose metabolism”, a pathway that was found to be enriched in influential metabolites in all three fluids and was also part of the “pentose phosphate pathway” that was enriched in influential metabolites in plasma. Along with fructose, glucose-6-phosphate is also involved in “starch (i.e., glycogen in animals) and sucrose metabolism”. This pathway was enriched in influential metabolites in both urine and amniotic fluid. However, the concentrations of all influential metabolites of this pathway varied differently in the two fluids between the two gestational stages. In urine, most of these metabolites were present at higher concentrations at 110 dg (glucose-6-phosphate, glucoronate and maltose), whereas only glycogen was present at a higher concentration in amniotic fluid at 110 dg.

Finally, guanidinoacetate is involved in the metabolic pathways of several amino acids. Six amino acids were found to be influential only in plasma (alanine, aspartate, glycine, leucine, serine and valine). Pathway enrichment analysis highlighted six pathways enriched in influential metabolites in plasma, including four related to amino acids: “aminoacyl-tRNA biosynthesis”, “glycine, serine and threonine metabolism”, “cyanoamino acid metabolism” and “cysteine and methionine metabolism”. In amniotic fluid, a pathway related to amino acids, “arginine and proline metabolism”, was also found to be enriched in influential metabolites.

**Table 1:** Enriched pathways in influential metabolites for OPLS-DA and in differential metabolites for mixed models.

Pathway	Method	Total metab.$^*$	Influential and/or differential metabolites
Alanine, aspartate and glutamate metabolism	Mixed models$^{p, u, af}$	24	2-Oxoglutarate$^{mm\_\{p,af\}}$, Alanine$^{mm\_\{p, u, af\}}$, Argininosuccinate$^{mm\_\{u\}}$, Asparagine$^{mm\_\{u\}}$, Aspartate$^{mm\_\{p, af\}}$, Glutamate$^{mm\_\{p, u\}}$, Glutamine$^{mm\_\{u, af\}}$,
Aminoacyl-tRNA biosynthesis	OPLS-DA$^p$ and mixed models$^{p, u, af}$	75	Alanine$^{opls\_{\{p\}};~mm\_\{p,u, af\}}$, Arginine$^{mm\_\{p, u, af\}}$, Asparagine$^{mm\_\{u\}}$, Aspartate$^{opls\_{\{p\}};~mm\_\{p,af\}}$, Cysteine$^{mm\_\{af\}}$, Glutamate$^{mm\_\{p, u\}}$, Glutamine$^{mm\_\{u, af\}}$, Glycine$^{opls\_{\{p\}};~mm\_\{p,u\}}$, Isoleucine$^{mm\_\{p\}}$, Leucine$^{opls\_{\{p\}};~mm\_\{p\}}$, Lysine$^{mm\_\{u, af\}}$, Proline$^{opls\_{\{p\}};~mm\_\{p, u, af\}}$, Serine$^{opls\_{\{p\}};~mm\_\{p,af\}}$, Threonine$^{mm\_\{p,u,af\}}$, Valine$^{opls\_{\{p\}};~mm\_\{p,af\}}$
Arginine and proline metabolism	OPLS-DA$^{af}$ and mixed models$^{p, u, af}$	77	5-Aminopentanoate$^{mm\_\{u, af\}}$, Arginine$^{mm\_\{p, u, af\}}$, Arginosuccinate$^{mm\_\{u\}}$, Aspartate$^{mm\_\{p, af\}}$, Citrulline$^{opls\_{\{af\}};~mm\_\{p, af\}}$, Creatine$^{opls\_{\{af\}};~mm\_\{p, af\}}$, Creatinine$^{opls\_{\{af\}};~mm\_\{p, u, af\}}$, Glutamate$^{mm\_\{p, u\}}$, Glutamine$^{opls\_{\{af\}};~mm\_\{u, af\}}$, Guanidinoacetate$^{opls\_{\{af\}};~mm\_\{p, u, af\}}$, Hydroxyproline$^{mm\_\{p\}}$, Proline$^{opls\_{\{af\}};~mm\_\{p, u, af\}}$, Pyroglutamate$^{mm\_\{u\}}$, Sarcosine$^{mm\_\{p, u\}}$ Spermidine$^{mm\_\{u, af\}}$
Ascorbate and aldarate metabolism	Mixed models$^{af}$	45	2-Oxoglutarate$^{mm\_\{af\}}$, Ascorbate$^{mm\_\{af\}}$, Glucuronate$^{mm\_\{af\}}$, Myo-inositol$^{mm\_\{af\}}$, Saccaric acid$^{mm\_\{af\}}$, Threonate$^{mm\_\{af\}}$
Cyanoamino acid metabolism	OPLS-DA$^p$	16	Aspartate$^{opls\_{\{p\}}}$, Glycine$^{opls\_{\{p\}}}$, Serine$^{opls\_{\{p\}}}$
Cysteine and methionine metabolism	OPLS-DA$^p$	56	Alanine$^{opls\_{\{p\}}}$, Aspartate$^{opls\_{\{p\}}}$, Serine$^{opls\_{\{p\}}}$
Galactose metabolism	OPLS-DA$^{p, u, af}$ and mixed models$^{p, u, af}$	41	Galactitol$^{opls\_{\{p, u\}};~mm\_\{p, u, af\}}$, Glucose$^{opls\_{\{p, af\}};~mm\_\{p, af\}}$, Glucose-6-phosphate$^{opls\_{\{p, u, af\}};~mm\_\{p, u, af\}}$, Glycerol$^{mm\_\{p, u, af\}}$, Lactose$^{opls\_{\{p, af\}};~mm\_\{p, af\}}$, Mannose$^{opls\_{\{p, af\}};~mm\_\{p, u, af\}}$, Myo-inositol$^{opls\_{\{p\}};~mm\_\{p, u, af\}}$, Sorbitol$^{opls\_{\{p, u\}};~mm\_\{p, u\}}$
Glutathione metabolism	Mixed models$^{u, af}$	38	Ascorbate$^{mm\_\{af\}}$, Cadaverine$^{mm\_\{u, af\}}$, Cysteine$^{mm\_\{af\}}$, Glutamate$^{mm\_\{u\}}$, Glycine$^{mm\_\{u\}}$, Oxidized glutathione$^{mm\_\{u, af\}}$, Pyroglutamate$^{mm\_\{u, af\}}$, Reduced glutathione$^{mm\_\{u, af\}}$, Spermidine$^{mm\_\{u,af\}}$
Glycine, serine and threonine metabolism	OPLS-DA$^p$ and mixed models$^{p, u, af}$	48	Aspartate$^{opls\_{\{p\}};~mm\_\{p, af\}}$, Betaine$^{opls\_{\{p\}};~mm\_\{p, u\}}$, Choline$^{mm\_\{p, u\}}$, Creatine$^{mm\_\{p, af\}}$, Cysteine$^{mm\_\{af\}}$, Glycerate$^{mm\_\{u, af\}}$, Glycine$^{opls\_{\{p\}};~mm\_\{p, u\}}$, Guanidinoacetate$^{opls\_{\{p\}};~mm\_\{p, u, af\}}$, Sarcosine$^{mm\_\{p, u\}}$, Serine$^{opls\_{\{p\}};~mm\_\{p, af\}}$, Threonine$^{mm\_\{p, u, af\}}$
Lysine biosynthesis	Mixed models$^{af}$	32	2-Aminoadipate$^{mm\_\{af\}}$, Aspartate$^{mm\_\{af\}}$, Lysine$^{mm\_\{af\}}$, 2-Oxoglutarate$^{mm\_\{af\}}$
Lysine degradation	Mixed models$^{u}$	47	2-Aminoadipate$^{mm\_\{u\}}$, 5-Aminopentanoate$^{mm\_\{u\}}$, Cadaverine$^{mm\_\{u\}}$, Glycine$^{mm\_\{u\}}$, Lysine$^{mm\_\{u\}}$
Pantothenate and CoA biosynthesis	Mixed models$^{p, af}$	27	Aspartate$^{mm\_\{p, af\}}$, 2-Oxoisovalerate$^{mm\_\{p\}}$, Cysteine$^{mm\_\{af\}}$ Pantothenate$^{mm\_\{p, af\}}$, Valine$^{mm\_\{p, af\}}$
Pentose phosphate pathway	OPLS-DA$^p$ and mixed models$^{af}$	32	Gluconate$^{opls\_{\{p\}};~mm\_\{af\}}$, Glucose$^{opls\_{\{p\}};~mm\_\{af\}}$, Glucose-6-phosphate$^{opls\_{\{p\}};~mm\_\{af\}}$, Glycerate$^{mm\_\{af\}}$
Starch and sucrose metabolism	OPLS-DA$^{u, af}$	50	Fructose$^{opls\_{\{u, af\}}}$, Glucose$^{opls\_{\{af\}}}$, Glucose-6-phosphate$^{opls\_{\{u, af\}}}$, Glucuronate$^{opls\_{\{u\}}}$, Glycogen$^{opls\_{\{af\}}}$, Maltose$^{opls\_{\{u\}}}$
Taurine and hypotaurine metabolism	Mixed models$^{af}$	20	Alanine$^{mm\_\{af\}}$, Cysteine$^{mm\_\{af\}}$, Hypotaurine$^{mm\_\{af\}}$, Taurine$^{mm\_\{af\}}$
Valine, leucine and isoleucine biosynthesis	Mixed models$^p$	27	2-Oxoisovalerate$^{mm\_\{p\}}$, Isoleucine$^{mm\_\{p\}}$, Leucine$^{mm\_\{p\}}$, Threonine$^{mm\_\{p\}}$, Valine$^{mm\_\{p\}}$

$^{<method>\_{\{<fluids>\}}}$ influential and/or differential metabolites for <method> ($^{opls}$ for OPLS-DA and $^{mm}$ for mixed models) in <fluids> (*p* for plasma, *u* for urine and *af* for amniotic fluid) $^*$ Total number of metabolites in the pathway.

### Differential analyses

Mixed linear models were fitted to each metabolite independently. The complete model involved two factors, gestational stage and fetal genotype, as well as their interaction (fixed effects), with sow as a random effect. In addition, all differential metabolites (i.e., metabolites for which the complete model was significantly better than the model with only the sow effect) were submitted to pathway enrichment analysis. To facilitate their individual interpretation, they were then associated with one of the best-fit sub-models derived from the complete model (see Methods). All the influential metabolites extracted by the multivariate analysis were also significantly differential in one of these mixed linear models, whatever the fluid. The detailed results of the mixed models are given in Supplementary Data S1.

The mixed models revealed that the metabolomes differed more between the two gestational stages than between genotypes in all three fluids. In plasma, 57 differential metabolites were associated with a model that included the effect of the stage gestation (complete, additive, and only stage models) whereas only 28 differential metabolites were found associated with a model that included the genotype effect (complete, additive, and only genotype models). In urine, the comparison identified 41 versus 20 metabolites and in amniotic fluid, 58 versus 6 metabolites (Table 2).

**Table 2:** Number of differential metabolites associated with each sub-model according to the fluid.

Sub-model	Plasma	Urine	Amniotic fluid
Complete	8	0	0
Additive	15	5	4
Only stage	34	36	54
Only genotype	5	15	2
Total for models with a stage effect	57	41	58
Total for models with a genotype effect	28	20	6
Total	62	56	60

#### Differences between stages of gestation

More differential metabolites were associated with a model with the stage of gestation effect (complete, additive and, only stage models) in plasma than in urine and amniotic fluid but, in amniotic fluid, 54 differential metabolites (out of 58) were associated with the model with only the stage effect, which is the highest number of differential metabolites for this model among the three fluids. In addition, temporal changes in the quantifications of metabolites associated with the only stage model differed more in plasma than in the other fluids. The majority of metabolites (28/34) were more concentrated at 110 dg than at 90 dg in plasma whereas only half the metabolites were more concentrated at 110 dg than at 90 dg in urine and amniotic fluid.

**Figure 2:**
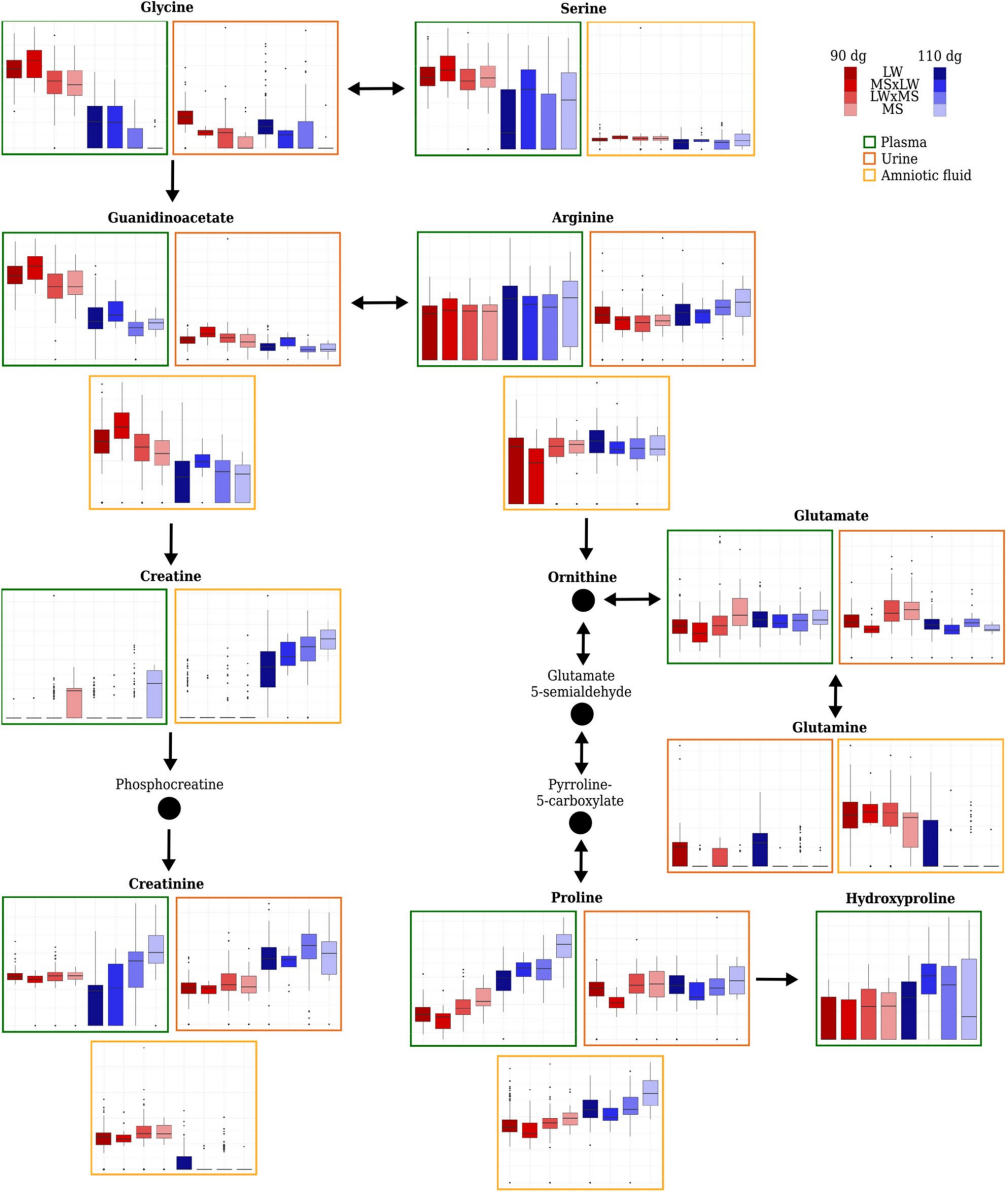
Relative concentrations of some metabolites involved in amino acid metabolism (“arginine and proline metabolism” and “glycine, serine and threonine metabolism”) in plasma, urine and amniotic fluid at the two stages of gestation (90 dg and 110 dg, in red and blue respectively) and fetal genotypes (LW, MS $\times$ LW, LW $\times$ MS and MS, from left to right respectively). For the sake of clarity, only nine and seven metabolites out of 15 in plasma and 15 differential metabolites in urine are shown. Metabolites in bold are those included in the **ASICS** reference library. The coordinates of the *y* axes in boxplots can not be compared between two metabolites because the relative concentration limits of the boxplots are adapted to each metabolite.

Among the 20 proteinogenic amino acids, 15 were differential in at least one fluid. All differential amino acids were associated with a stage effect model, except for five: in urine, alanine, glutamine, glycine, proline and threonine, which were associated with the only genotype model. These 15 amino acids are involved in four pathways that were enriched in differential metabolites in all three fluids (Table 1), “alanine, aspartate and glutamate metabolism”, “aminoacyl-tRNA biosyntheis”, “arginine and proline metabolism” and “glycine, serine and threonine metabolism”. Fourteen out of 20 metabolites in plasma and 11 out of 18 metabolites in amniotic fluid were more concentrated at 110 dg (see Fig. 2 for a representation of arginine, creatine, creatinine, glutamine, guanidinoacetate, proline and serine).

However, differences were also identified in the three fluids. In plasma, 2-oxoisovalerate, isoleucine, leucine, threonine, and valine were all differential and associated with the only stage model. They are involved in the “valine, leucine and isoleucine biosynthesis”, a pathway related to amino acids that was enriched in plasma. In amniotic fluid, 2-aminoadipate, aspartate, lysine, and 2-oxoglutarate were differential and were all associated with the only stage model. They are involved in another amino acid related pathway, “lysine biosynthesis”, which was enriched in amniotic fluid. These four metabolites (lysine, valine, leucine, and isoleucine) are described as essential amino acids in humans and in pigs and it is widely accepted that they are not synthesized by these organisms. However, metabolites of the two pathways (“valine, leucine and isoleucine biosynthesis” and “lysine biosynthesis” pathways) were all significantly more concentrated at 110 dg, which explains why they were found in our study.

In urine, fewer differential metabolites were associated with a model with the stage effect than in the other two fluids, especially amino acids. However, “galactose metabolism”, which was enriched in differential metabolites in the urine, contained five metabolites (myo-inositol, glucose-6-phosphate, mannose, sorbitol and galactitol), that were all associated with the only stage model.

Finally, four differential metabolites were also found in amniotic fluid, associated with a model including the stage effect: glucose-6-phosphate, gluconate, glucose and glycerate, among which three out of four (i.e., except for gluconate) were associated with the only stage model. These metabolites are all involved in the “pentose phosphate pathway” which was found to be enriched in differential metabolites in amniotic fluid. This pathway was previously found to be enriched in influential metabolites (as obtained by OPLS-DA) but in plasma rather than in amniotic fluid. In addition, two metabolites of this pathway, glucose, and gluconate, varied in opposite directions in the two fluids: glucose concentration was higher at 110 dg in plasma whereas it was higher at 90 dg in amniotic fluid (while the reverse was true for the concentration of gluconate).

#### Differences between genotypes

More differential metabolites were associated with a model that included the genotype effect (complete, additive and only genotype models) in plasma than in urine and amniotic fluid (28 metabolites compared to 20 and 6, respectively; Table 2).

**Figure 3:**
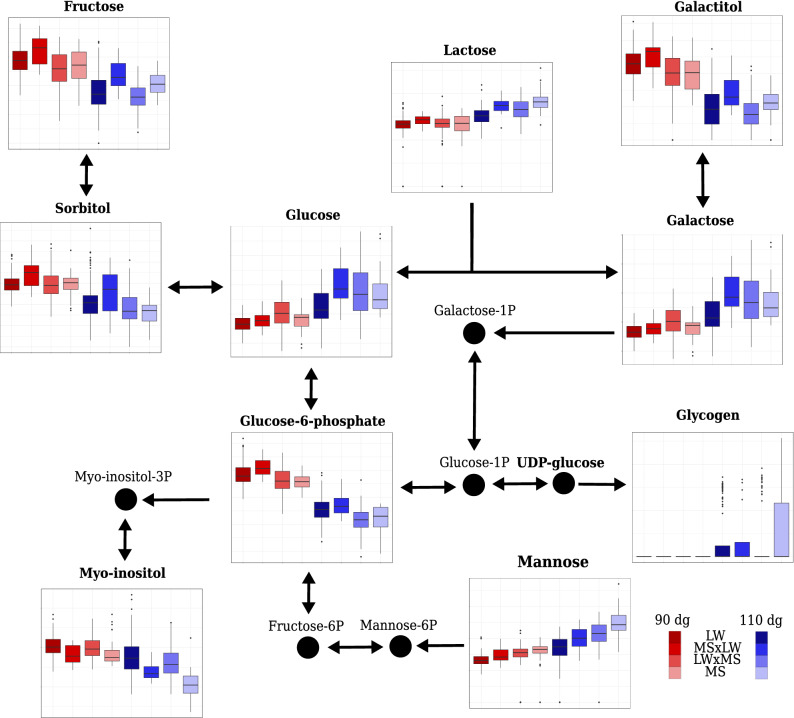
Relative concentrations of some metabolites involved in the carbohydrate metabolism pathways (“galactose metabolism” and “starch and sucrose metabolism”) in plasma according to the stage of gestation (90 dg and 110 dg, in red and blue respectively) and fetal genotypes (LW, MS $\times$ LW, LW $\times$ MS and MS, from left to right, respectively). Metabolites in bold are those included in the **ASICS** reference library. The coordinates of the *y* axes in boxplots cannot be compared between two metabolites because the relative concentration limits of the boxplots are adapted to each metabolite.

In plasma, six differential metabolites (galactitol, glucose, glucose-6-phosphate, mannose, myo-inositol, and sorbitol) were associated with the complete or the additive model, which included a genotype effect, and two (glycerol and lactose) with the only stage model. Among these eight metabolites, some were also differential in urine and amniotic fluid but were not usually associated with a model including the genotype effect in these fluids. The only exceptions were glycerol in urine (associated with the only genotype model) and the galactitol in amniotic fluid (also associated with the only genotype model). In addition, these eight metabolites are all involved in the “galactose metabolism” pathway, which was enriched in differential metabolites in all fluids (Fig. 3 for plasma and Supplementary Figs. S4 and S5 for urine and amniotic fluid, respectively). In plasma, mannose and glucose were more concentrated at 110 dg than at 90 dg and were also more concentrated in MS than in LW at both 90 dg and 110 dg. On the contrary, the other three metabolites (glucose-6-phosphate, sorbitol and myo-inositol) were more concentrated at 90 dg and 110 dg in LW and the galactitol was more concentrated in MS at 110 dg. In addition, the concentration of myo-inositol was higher when the fetus had a LW father (whatever the mother genotype) and the concentration of glucose-6-phosphate, sorbitol and galactitol was higher when the fetus had a MS father. Conversely, in urine, the concentration of glycerol was higher when the fetus had a LW mother.

In plasma and urine, 12 differential metabolites of the enriched “arginine and proline metabolism” and “glycine, serine and threonine metabolism” pathways were also associated with a model including the genotype effect: creatinine (complete model), aspartate, glycine, guanidinoacetate and proline (additive model), choline and creatine (only genotype model) in plasma and glutamate and guanidinoacetate (additive model), 5-aminopentanoate, glutamine, glycerate, glycine, proline and threonine (only genotype model) in urine (see Fig. 2 for a representation of creatine, creatinine, glutamate, glutamine, glycine, guanidinoacetate and proline). Among these metabolites, six were more concentrated in MS than in LW (5-aminopentanoate and glycerate in urine, aspartate, choline and creatine in plasma and proline in both urine and plasma). The concentrations of aspartate in plasma and of glycerate in urine were also higher when the fetus had a MS father compared to a LW father (paternal effect; Supplementary Fig. S6), while median concentrations of choline and creatine in plasma were higher than 0 only in fetuses with both a MS mother and father (effect of the pure MS genotype). Three metabolites (glutamine in urine and glycine and guanidinoacetate in plasma and urine) were more concentrated in LW than in MS.

**Figure 4:**
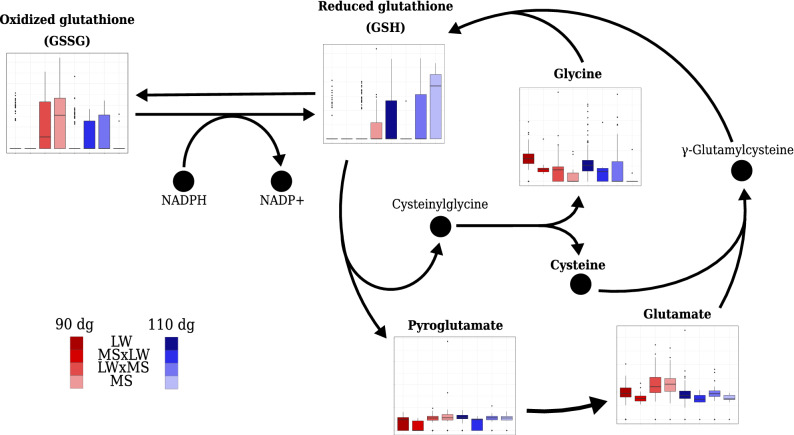
Relative concentrations in urine according to the stage of gestation (90 dg and 110 dg, in red and blue respectively) and the fetal genotype (LW, MS $\times$ LW, LW $\times$ MS and MS, from left to right) for some metabolites in the “glutathione pathway”. For the sake of clarity, only the $\gamma$-glutamyl-cycle is represented in this figure. Metabolites in bold are those included in the **ASICS** reference library. The coordinates of the *y* axes in boxplots can not be compared between two metabolites because relative concentration limits of the boxplots are adapted to each metabolite.

In urine, four differential metabolites associated with the enriched “glutathione pathway” (Fig. 4) were associated with the only genotype model (oxidized glutathione, glycine, and pyroglutamate) and one was associated with the additive model (glutamate). These metabolites were more concentrated in LW than in MS fetuses at 110 dg and more concentrated in MS than in LW at 90 dg, except glycine, which was still more concentrated in LW than in MS at 90 dg.

## Discussion

The biological processes of fetal maturation and fetal growth retardation are of major interest in humans and in several mammalian livestock species, including sheep^15^ and pig^16,17^. These processes are related to fetal development during late gestation, which is difficult to explore in mammalian species due to the invasiveness of experiments performed during that period. Since impaired maturation may induce postnatal developmental delay, metabolic syndrome, or early death^18^, the level of development at birth is currently evaluated by measuring birth weight^3^ as a proxy for intrauterine development. The study of the metabolome in the late gestation period is a promising way to predict immediate or later outcomes as well as to evaluate fetal growth retardation^19^.

In humans, some metabolomic studies have already been performed on amniotic fluid collected during amniocentesis in the second or the third trimester of pregnancy^20,21^. However, most other metabolomic surveys in humans have been performed later at birth, especially on cord blood^22–24^, or on urine^25^. In non-human mammalian species, only a few studies related to the metabolome during late developmental processes have been published so far, including one plasmatic NMR study on pig fetuses in late gestation^26^.

Using NMR techniques, we acquired untargeted metabolomic measurements on three fluids (plasma, urine, and amniotic fluid) in 611 pig fetuses from four different genotypes at two different gestational stages. It should be noted that one limit of this technique is that it is not appropriate for quantifying lipids that form a heap of peaks in NMR spectra in water soluble fluids such that amniotic fluid, plasma and urine. No other lipidiomic approach has been investigated in this study. In addition, only a small number of metabolites involved in the glycolysis pathway were found in our study because most were not present in the **ASICS** reference library. Hence, the “glycolysis pathway” and the lipid related metabolisms were not found in our study, but are known to be important in late gestation. For example, Fainberg et al.^27^ investigated the lipidome of hepatic tissues to compare MS and commercial piglets immediately after birth. They identified five fatty acids that differentiated MS from commercial breeds and suggested that these differences may explain the better adaptability of MS piglets to the energetic demand for thermoregulation.

Despite these limitations, major differences were found in the three fluids at the two gestational stages pointing to a dramatic change in fetal metabolism between 90 and 110 dg. Such a metabolic switch was also recently reported in the metabolome of the amniotic fluid in humans between the second and the third trimesters of pregnancy^28^. The metabolic switch observed in the present study is also consistent with our previous findings obtained using the same experimental design that highlighted important variations in the muscle and intestinal transcriptomes and in the muscle and adipose tissue proteomes in a smaller subset of the fetuses of both breeds^10–13^. More precisely, the first study of Voillet et al.^10^ identified important changes in the muscle transcriptome of both breeds between the two gestational ages (90 and 110 dg) by focusing on interaction effects between breed and gestational age. Notably, the study demonstrated that genes involved in muscular development are up-regulated around 90 dg and genes linked to metabolic functions, like gluconeogenesis, are up-regulated at 110 dg, whatever the genotype. Other later studies^11–13^ confirmed this finding using similar analyses of the intestinal transcriptome and of the muscle and adipose tissue proteomes.

The present study identified the metabolomic pathways involved in the regulation of carbohydrates, amino acids, and glutathione metabolisms, which were found to be enriched in influential or differential metabolites. Many of the metabolites we identified are directly related to cellular energy levels and metabolism. It is indeed critical that carbohydrate metabolism is efficient at birth to provide the newborn piglet with the energy needed to overcome hypothermia due to birth, and subsequently with the energy required for maintenance, thermoregulation and growth^2,29^. In addition, the different pathways related to carbohydrate metabolisms have been shown to be altered during fetal development in neonates with IUGR^30^. In our study, “galactose metabolism” was the only pathway enriched in influential or differential metabolites whatever the fluid or the statistical method used. This pathway is essential in mammalian species, especially during fetal and neonatal development, because it plays an important role in energy delivery^31^. All seven metabolites (galactitol, glucose, glucose-6-phosphate, lactose, mannose, sorbitol, and myo-inositol) included in the “galactose metabolism” were identified by **ASICS** in the three fluids.

Among the metabolites identified in the “galactose metabolism” pathway, the concentration of myo-inositol has already been proposed as a marker of the development of obesity and type 2 diabetes in human adults^25,32^ and as a marker of IUGR in both humans^33,34^ and pigs^26^. In these studies, a higher concentration of myo-inositol in plasma or urine was associated with a higher risk of IUGR and, thus, with lower maturity. In pigs, NMR metabolomic profiling performed on fetuses at 110 days of gestation demonstrated that low-weight fetuses had higher concentrations of myo-inositol in the plasma than high-weight fetuses^26^. Dessì and Fanos^33^ suggested that, in fetuses with IUGR, higher concentrations of myo-inositol in the plasma may reflect altered glucose metabolism and showed that fetuses with IUGR were also associated with a decrease in lipid synthesis and cell proliferation due to the reduction in insulin secretion. Such effects lead to lower birth weight. Consistently, we observed lower plasma concentrations of myo-inositol in MS fetuses, considered as more mature, at both 90 and 110 dg, despite the lower concentration of myo-inositol in urine at 90 dg compared to 110 dg (no genotype effect in this fluid). This finding suggests that more efficient glucose metabolism may partly explain the greater maturity at birth of MS piglets compared to LW piglets.

Glucose, another metabolite, is essential for the provision of the energy required for fetal growth and development. The concentration of glucose in pig plasma has already been shown to be lower in IUGR than in non-IUGR newborns^35,36^. In our study, glucose was indeed differential and more concentrated in MS than in LW, both at 90 and 110 dg. Therefore, both myo-inositol (less concentrated in MS) and glucose (more concentrated in MS) may partly explain the better maturity of MS at birth. Moreover, the concentrations of these two metabolites were more influenced by the paternal genotype, which is consistent with a parental imprinting mechanism^37,38^. The role of genes, such as *IGF2* under parental imprinting during gestation has already been described^39^ and its role in the fetal glycogen synthesis has also been demonstrated^40^. However, the parental imprinting phenomenon has never previously been studied using metabolomic data before the current study in which we demonstrated that the concentration of some metabolites (e.g., myo-inositol and glucose) depends on the paternal or maternal genotype in the reciprocal crossed fetuses.

In addition, during the last month of gestation, glucose is stored in fetal tissues, particularly in muscle and liver, in its polymerized form, i.e., glycogen. The storage of glycogen just before birth has been known since centuries^41^: comparison of glycogen contents at birth and a few hours after birth showed that muscle and liver glycogen contents dropped dramatically in mammalian species a few hours after birth. In our study, glycogen was detected in urine, amniotic fluid, and to a lesser extent, in plasma, and its concentration was significantly higher at 110 dg than at 90 dg in all three fluids. At birth, piglets mainly rely on glycogen as an energy-yielding substrate before colostrum consumption^42,43^. Studying pig fetuses close to term in relation to their value for survival at birth, Leenhouwers et al.^4^ and Voillet et al.^11^ showed that glycogen content in liver and muscle increased with increased chance of survival. Since glycogen is a multibranched polysaccharide of glucose described as a reserve in tissues, it was surprising to find it in the three fluids we studied (i.e., plasma, urine, and amniotic fluid). One possible explanation is that, as glycogen synthesis in tissues is intense, some polymers of glucose may have been released into the fluids just before birth.

Concerning carbohydrates, many amino acids were highlighted in our analyses. Nine amino acid metabolism pathways were found to be enriched in influential and differential metabolites at the end of gestation: “alanine, aspartate and glutamate metabolism”, “aminoacyl tRNA biosynthesis”, “arginine and proline metabolism”, “cyanoamino acid metabolism”, “cysteine and methionine metabolism”, “glycine, serine and threonine metabolism”, “lysine biosynthesis”, “lysine degradation” and “valine, leucine and isoleucine biosynthesis”. They all respond to the need for fetal development and maturation since amino acids play nutritional, physiological and regulatory roles. Twenty amino acids are known to be involved in these pathways^44^. In our study, these pathways were enriched in 15 differential amino acids, including five essential amino acids (i.e., amino acids that cannot be synthetized by animals) and 10 non essential amino acids (i.e., that can be synthetized by animals). Amino acids of the “arginine and proline metabolism” (arginine, asparagine, aspartate, glutamate, glutamine, ornithine, and proline) are already well studied during gestation because of their essential role in fetal growth and development both in humans and pigs^45^.

The arginine concentration in the amniotic fluid in early pregnancy has been described as being positively correlated with birth weight, body length and head circumference of babies^46^. In addition, Wu et al.^47^ showed that arginine supplementation of sows during gestation reduced the stillbirth rate and the risk of IUGR. These two studies support an important role of arginine in fetal maturation and their results are also consistent with ours: arginine was identified by mixed models as differential. It was more concentrated at 110 dg than at 90 dg in all three fluids, although an earlier study^35^ showed a reduction in the concentration of arginine in plasma between 90 and 110 dg.

Like arginine, glutamine is also highly concentrated in amniotic fluid mainly in early gestation^48^. At the end of gestation, the amniotic fluid serves as a nutritional reservoir for the fetus and, as a result, uptake of glutamine by the fetus may reduce the concentration of glutamine in amniotic fluid^45^. Hence, the glutamine concentration is usually considered as a limiting factor of fetal growth and a lower concentration is known to be associated with an increased risk of IUGR risk. This was confirmed by our study in which glutamine was not found at 110 dg in amniotic fluid. Proline, which is also involved in the “arginine and proline metabolism” pathway, is less frequently used in sow nutrition than arginine and glutamine^49^. However, its important role in polyamine synthesis during the pig gestation has already been demonstrated^50^. As expected, the higher concentration of proline in MS than in LW at 90 and 110 dg in plasma could be related to the lower maturity of LW piglets and to a potential delay in development already identified in these fetuses^10–13^. In addition, in plasma, crossbred fetuses have intermediate concentrations of proline, with no specific maternal or paternal effect.

Serine, glycine and guanidinoacetate metabolites are involved in the “one-carbon metabolism” (this metabolism is not a KEGG pathway and its enrichment was consequently not analyzed here). This metabolism is involved in DNA methylation by providing methyl groups^45^. Like for imprinting genes, DNA methylation is an important epigenetic mechanism of fetal gestation. Previous studies have shown the association between IUGR and epigenetic alterations^45^. Lin *et al.*^35^ also showed that the concentration of serine significantly decreased between 90 dg and 110 dg in pigs. A higher concentration of serine and glycine in plasma has also been reported in IUGR rat fetuses compared to normal weight fetuses^51^. This is consistent with our findings: glycine and serine concentrations were differential and in plasma a higher concentration was found at 90 dg than at 110 dg. The concentration of glycine was also more concentrated in LW than in MS at both 90 and 110 dg. Guanidinoacetate exhibited a maternal effect: concentrations of this metabolite in fetuses with a LW mother were higher than in fetuses with a MS mother whatever the stage of gestation. For glycine, the same maternal effect was observed at 90 dg. These metabolites are precursors of creatine (see Fig. 2), which is known to be involved in energy metabolism and development of skeletal muscles^52,53^. In plasma, both creatine and creatinine were differential and were more concentrated in MS than in LW at 110 dg. In contrast to our findings, other studies on IUGR reported a higher concentration of creatine and creatinine in IUGR fetal pigs^35^ or newborns^25^. However, in our study, it should be noted that the concentration of creatinine in plasma changed differently according to the genotype. Indeed, at 90 dg, the concentration was approximately the same, whatever the genotype, but in LW, the concentration then decreased sharply whereas in MS, it increased sharply and was higher at 110 dg than at 90 dg.

As the production of oxidants increases during gestation due to cell proliferation, it is necessary that the “glutathione metabolism” is efficient because it plays a role in oxidative defense^54,55^. An increase in oxidative stress has already been associated with IUGR or preterm infants^55–58^. As expected, in our study, “glutathione metabolism” was enriched in differential metabolites in urine. Reduced glutathione is formed from glutamate, cysteine, and glycine and protects cells against oxidative damage by removing hydrogen peroxide^44^. Oxidized glutathione was only detected in MS and was more concentrated at 90 dg than at 110 dg. Conversely, reduced glutathione was more concentrated at 110 dg than at 90 dg in MS and was almost undetectable in LW. Taken together, these results suggest a better oxidative defense in MS than in LW. Interestingly, the concentration of pyroglutamate, another metabolite involved in the “glutathione metabolism”, has already been shown to be more concentrated in the plasma of IUGR fetuses than in a normal birth weight group, likely due to reduced glutathione synthesis^59^. In addition, pyroglutamate has been suggested as a biomarker for IUGR in fetal plasma^35^. However, this is still not clearly established fact. For instance, Jackson et al.^58^ showed that the pyroglutamate/creatinine ratio in urinary excretion just after birth was higher in preterm infants. We observed the same trend during late gestation: pyroglutamate in plasma was more concentrated at 110 dg than at 90 dg and the pyroglutamate/creatinine ratio was higher in LW than in MS in urine.

## Conclusions

Our study of changes in metabolism in late gestation in two contrasted pig breeds provide useful insights into potential biomarkers and metabolism pathways associated with survival at birth. In particular, proline and myo-inositol are two promising metabolites for the characterization of piglet maturity. They illustrate the importance of amino acid and carbohydrate metabolisms for fetal development in late gestation.

However, the relative quantification of metabolites we used in this study might not be sufficient to derive biomarkers with thresholds based on absolute quantifications. To achieve this goal, other targeted studies will be necessary, along with the training of an adequate prediction method to set appropriate thresholds. The comprehensive view of fetal metabolome we provided paves the way for the design of such studies.

## Methods

### Animals and plasma, urine and amniotic fluid sampling

Plasma, urine, and amniotic fluid samples were obtained from 611 pig fetuses at two gestational stages (90 and 110 days, average gestation term 114 days). MS and LW sows were inseminated with mixed semen (LW and MS) so that most litters were composed of purebred fetuses (LW or MS) and crossbred fetuses (LW $\times$ MS from MS sows and MS $\times$ LW from LW sows). MS and LW breeds were chosen as two extreme breeds for piglet mortality at birth, a better survival rate being observed in MS piglets. The experimental design is described in detail in Voillet et al.^10^  and is summarized in Supplementary Fig. S7. A total of 329 fetuses had a LW mother and 282 fetuses had a MS mother. The fetuses were obtained by caesarean section. Fetuses were weighed (statistics on weights are provided in Table 3), and on average, LW weighed more than MS despite their lower maturity.

**Table 3:** Fetus weights at 90 and 110 days of gestation according to genotype (mean ± standard deviation in grams).

Genotype	90 days of gestation	110 days of gestation
LW	619 ± 141	1171 ± 323
MS $\times$ LW	633 ± 91	1292 ± 197
LW $\times$ MS	579 ± 114	1092 ± 201
MS	490 ± 86	910 ± 101

After laparotomy of the sow, blood (approximately 5 mL) was collected individually from the umbilical artery of the piglets using a 21-gauge needle and a 5 mL syringe and placed in heparinized tubes. After section of the umbilical cord, the fetus was euthanized^13^. Plasma was prepared by low-speed centrifugation (2000*g* for 10 min at $4\,^\circ$C) and stored at $-80\,^\circ$C until further analysis. The amniotic fluid (10 mL) was collected during the caesarean and immediately centrifuged (2000*g* for 10 min at $4\,^\circ$C) to discard cell debris and stored at $-20\,^\circ$C until further analysis. The urine samples were collected directly in the bladder with a 5 mL syringe during dissection of the fetuses, immediately frozen to avoid contamination and stored at $-80\,^\circ$C until further analysis.

### Nuclear magnetic resonance

The detailed protocol for sample preparation, spectra acquisition and preprocessing can be found in Lefort et al.^60^. Briefly, each sample of plasma and amniotic fluid (200 $\upmu$L) was diluted in 500 $\upmu$L deuterated water (D$_2$O) and centrifuged without the addition of internal standard to improve spectra quality. For urine, 200 $\upmu$L of phosphate buffer prepared in deuterated water (0.2 M, pH 7.0) were added to 500 $\upmu$L of urine, vortexed, centrifuged at 5000*g* for 15 min, and 600 $\upmu$L transferred to 5 mm NMR tube. All $^1$H NMR spectra were acquired on a Bruker Avance DRX-600 spectrometer (Bruker SA, Wissembourg, France) operating at 600.13 MHz for $^1$H resonance frequency and at 300 K using the Carr-Purcell-Meiboom-Gill (CPMG) spin-echo pulse sequence. The Fourier transformation was calculated on 64,000 points. All spectra were phased, baseline corrected and then calibrated on the resonance of lactate (1.33 ppm) using Topspin (V2.1, Bruker, Biospin, Munich, Germany). The regions corresponding to water resonance (5.1–4.5 ppm) and urea (6.5–6.0 ppm) were excluded to eliminate artefacts of residual water and urea.

### Metabolite identification and quantification

To measure the concentration of metabolites in the three fluids, NMR metabolomic spectra were processed with **ASICS**, a recently developed R package^60^. Before quantification, spectra were normalized by the area under the curve and aligned with preprocessing functions available in the Bioconductor R package **ASICS**^60^ (version 2.0.0). The metabolites in all fluids were identified and quantified using the **ASICS** method available in the same package. Quantification was performed using the default reference library provided in the package and was processed independently for each fluid but the same maximum chemical shift, set at 0.02, was allowed for each. The library alignment was improved (compared to that described in Lefort et al.^60^) by using a global quality control criterion: the correlation between quantifications and targeted buckets of the spectra was maximized to choose the best alignment between peaks. Finally, metabolites that had at least 25% of quantifications larger than 0 in at least one condition (stages of gestation and genotypes) were retained. The others were removed from the list of identified metabolites (quantification set to 0).

Note that the **ASICS** quantification method is threshold-based, meaning the estimation of the quantification is exactly 0 for some metabolites. In all cases, it means that the peaks corresponding to these metabolites are below the noise level in the corresponding complex mixture spectra. But, as a consequence, a threshold effect is visible in some of the boxplots of Figs. 2 and 3, where some quantification distributions are represented by a flat horizontal line centered on zero. However, the effect of such a threshold is negligible because, in all cases, it means that the real concentration, if not really zero, is so low that it can not be distinguished from noise in the complex spectra.

### Spectra quality control

Plasma relative quantifications were previously validated in Lefort et al.^60^ using biochemical targeted dosages of three metabolites (glucose, fructose and lactate) in a subset of the samples. The results of a an Orthogonal Projections to Latent Structures Discriminant Analysis^14^ (OPLS-DA) based on the standard bucket approach were compared with the results of an OPLS-DA based on metabolite quantifications. The comparison showed good reproducibility and similar discriminative power between conditions for the bucket and quantification approaches, insuring minimum loss of information during quantification preprocessing.

Principal Component Analyses (PCA) was used to detect potential outliers and batch effects due to experimental covariates: sex, breeding batch, and sow. All plots are shown in Supplementary Figs. S8–S10. PCA did not identify any sex, batch or experimental effect but a sow effect was clearly visible and, whenever possible was included in the subsequent analyses.

### Multivariate exploratory analyses

All statistical analyses were performed with R (version 3.6.0)^61^. The effect on the metabolome of the stage of gestation (i.e., 90 dg and 110 dg) was first investigated with OPLS-DA^14^. Three OPLS-DA were performed independently on each fluid to identify the metabolites with the highest discriminant power between the two gestational stages: the most influential metabolites, i.e., metabolites with a VIP index greater than 1, were extracted. The relevance of the results was insured by estimating the predictive power of each model with a 10-fold cross-validation error.

### Univariate differential analyses with mixed models

As OPLS-DA was limited to the study of one factor with only two levels, we completed it with more complete analyses based on mixed models that can incorporate multiple effects, including the random effect of the sow used as a proxy for the effect of the uterine environment. This effect must not be mistaken with a parental effect originating from the genotype. Mixed models were used to identify metabolites with differential concentrations between conditions (gestational stages and genotypes), by fitting the following model for each fluid and each metabolite:

1 $$\begin{aligned} y_{ijk} = \mu + A_i + FG_j + I_{ij} + S_k + \epsilon _{ijk} \end{aligned}$$

with $y_{ijk}$ the vector of metabolite concentrations for gestational stage *i* ($i \in \{\text {d90}, \text {d110}\}$), genotype *j* ($j \in \{\text {LW}, \text {LW}\times \text {MS}, \text {MS}\times \text {LW}, \text {MS} \}$) and mother (sow) *k*. In this model, $\mu$ is the mean effect, $A_i$ the fixed effect of the gestational stage, $FG_j$ is the fixed effect of the genotype, $I_{ij}$ is the effect of the interaction between the gestational stage and the genotype, $S_k \sim \mathcal {N}(0, \sigma _r^2)$ is the random effect of the sow and $\epsilon _{ijk} \sim \mathcal {N}(0, \sigma _e^2)$ is a noise term.

For all metabolites, this model was tested against the model with only the sow effect ($y_{ijk} = \mu + S_k + \epsilon _{ijk}$) with a Fisher’s test. *p* values were then adjusted with the Benjamini and Hochberg (FDR) correction^62^. Finally, using the same methodology as Voillet et al.^10^, each differential metabolite (i.e., the metabolites with an adjusted *p* value of less than 0.05) was associated with one of the following sub-models:

- complete: $$y_{ijk} = \mu + A_i + FG_j + I_{ij} + S_k + \epsilon _{ijk}$$
- additive: $$y_{ijk} = \mu + A_i + FG_j + S_k + \epsilon _{ijk}$$
- only stage: $$y_{ijk} = \mu + A_i + S_k + \epsilon _{ijk}$$
- only genotype: $$y_{ijk} = \mu + FG_j + S_k + \epsilon _{ijk}$$

In contrast to the approach that would have consisted in independently testing each effect of the complete model (stage effect, genotype effect and interaction), selecting the best fit sub-model avoids overfitting and produces the best set of relevant effects for each metabolite. Since the four models described above are not nested (the “only stage” and “only genotype” models are not), this selection cannot be performed using a standard Fisher’s test so we used a model selection approach instead and selected the model with the minimum Bayesian Information Criterion (BIC)^63^.

### Pathway enrichment

Pathway enrichment analysis was performed with the web-based tool suite MetaboAnalyst^64^ (version 4.0) with the MetPA module^65^. *Sus scrofa* pathways were not available in MetaboAnalyst, so the *Homo sapiens* KEGG pathways were used, as a reference instead. We also checked the differences between human and pig pathways on the KEGG database and the two pathways were found to be almost identical. This confirmed the relevance of using the human pathways in MetaboAnalyst. Finally, hypergeometric tests were performed to extract pathways enriched in influential or differential metabolites and *p* values were corrected for multiple testing using the Benjamini and Hochberg approach. This analysis was carried out for each fluid and on OPLS-DA and mixed model results independently.

### Ethics statement

All the fluids from pig fetuses were obtained in the framework of the PORCINET project (ANR-09-GENM-005-01, 2010–2015). The experiment authorization number of the experimental farm GenESI (Pig phenotyping and Innovative breeding facility, 10.15454/1.5572415481185847E12) is A-17-661. The procedures and the animal management complied with European Union legislation (Directive 2010/63/EU) and the French legislation in the Midi-Pyrénées Region (Decree 2001-464). All experiments were performed in accordance with relevant guidelines and regulations and were approved by the ethical committee of the Midi-Pyrénées Regional Council (authorization MP/01/01/01/11).

## Supplementary information

Supplementary Information 1

Supplementary Information 2

## Data Availability

The data supporting the results of this article are available in the MetaboLights database^66^: MTBLS1541 (www.ebi.ac.uk/metabolights/MTBLS1541).
