# Supplementary material for: The maturity in fetal pigs using a multi-fluid metabolomic approach
Source: Sci Rep. 2020 Nov 16;10:19912. doi: 10.1038/s41598-020-76709-8 (PMC7670440; doi:10.1038/s41598-020-76709-8)
Supplement: Supplementary file 1 — Supplementary Information 1 [file 41598_2020_76709_MOESM1_ESM.pdf]

Supplementary material of the article "The maturity in fetal pigs using a multi-fluid metabolomic approach"

Gaëlle Lefort[1,2], Rémi Servien[3,4], Hélène Quesnel[5], Yvon Billon[6], Laurianne Canario[2], Nathalie Iannuccelli[2], Cécile Canlet[7], Alain Paris[8], Nathalie Vialaneix[1,+], Laurence Liaubet[2,+]

[1]*INRAE, UR875 Mathématiques et Informatique Appliquées Toulouse, F-31326 Castanet-Tolosan, France*
[2]*GenPhySE, Université de Toulouse, INRAE, ENVT, F-31326, Castanet-Tolosan, France*
[3]*INRAE, Univ. Montpellier, LBE, 102 Avenue des étangs, F-11000 Narbonne, France*
[4]*INTHERES, Université de Toulouse, INRAE, ENVT, Toulouse, France*
[5]*PEGASE, INRAE, Institut Agro, 35590, Saint Gilles, France*
[6]*INRAE, GENESI, F-17700, Saint Pierre d'Amilly, France*
[7]*Axiom Platform, MetaToul-MetaboHUB, National Infrastructure for Metabolomics and Fluxomics, Toulouse, France*
[8]*MCAM, Muséum National d'Histoire Naturelle, CNRS, Paris, France*
[+]*these authors contributed equally to this work*
{gaelle.lefort,laurence.liaubet}@inrae.fr

S1 Supplementary tables

Table S1. Metabolites identified in each fluid.

metabolite names in ASICS	alternative names	plasma	urine	amniotic fluid
1,3-Diaminopropane		X	X	
2-AminoAdipicAcid	2-Aminoadipate	X	X	X
2-AminobutyricAcid		X		
2-Deoxycytidine			X	X
2-Oxoglutarate		X		X
2-Oxoisovalerate		X		
3-Hydroxybutyrate			X	
3-Methyl-L-Histidine			X	X
3-MethyladipicAcid			X	
4-AminoHippuricAcid		X		
4-HydroxyphenylAceticAcid		X		
5-AminoValericAcid	5-Aminopentanoate		X	X
alpha-HydroxyisobutyricAcid				X
ArgininosuccinicAcid	Arginosuccinate		X	
AscorbicAcid	Ascorbate			X
Azelaic Acid				X
Betaine		X	X	X
Cadaverine		X	X	X
CholineChloride	Choline	X	X	
Citrate				X
Creatine		X		X
Creatinine		X	X	X
D-Fructose	Fructose	X	X	X
D-Fucose		X	X	X
D-Galactose				X
D-GluconicAcid	Gluconate	X	X	X
D-Glucose	Glucose	X	X	X
D-Glucose-6-Phosphate	Glucose-6-phosphate	X	X	X
D-GlucuronicAcid	Glucuronate	X	X	X
D-Maltose	Maltose		X	
D-Mannose	Mannose	X	X	X
D-Sorbitol	Sorbitol	X	X	X
DehydroAscorbicAcid		X	X	X
Ethanolamine		X	X	X
EthylmalonicAcid		X		
Galactitol		X	X	X
GlycericAcid	Glycerate		X	X
Glycerol		X	X	X
Glycerophosphocholine		X		X
Glycogen		X	X	X
GuanidinoaceticAcid	Guanidinoacetate	X	X	X
Hypotaurine				X
IsocitricAcid		X	X	X
IsovalericAcid		X		
L-Alanine	Alanine	X	X	X
L-Arabitol		X	X	X
L-Arginine	Arginine	X	X	X
L-Asparagine	Asparagine		X	
L-Aspartate	Aspartate	X	X	X
L-Carnitine		X		

Table S1 continued from previous page

metabolite names in ASICS	alternative names	plasma	urine	amniotic fluid
L-Citrulline	Citrulline	X	X	X
L-Cysteine	Cysteine			X
L-Cystine		X	X	X
L-GlutamicAcid	Glutamate	X	X	X
L-Glutamine	Glutamine		X	X
L-Glutathione-oxidized	Oxidized glutathione		X	X
L-Glutathione-reduced	Reduced glutathione		X	X
L-Glycine	Glycine	X	X	X
L-Isoleucine	Isoleucine	X		X
L-Leucine	Leucine	X		X
L-Lysine	Lysine		X	X
L-Proline	Proline	X	X	X
L-Serine	Serine	X	X	X
L-Threonine	Threonine	X	X	X
L-Valine	Valine	X		X
Lactate		X	X	X
Lactose		X	X	X
Levoglucosan			X	X
MandelicAcid		X		
Methanol			X	X
Methylguanidine			X	
Myo-Inositol		X	X	X
N-Acetylglycine		X	X	
PantothenicAcid	Pantothenate	X		X
Phenethylamine		X		
PropyleneGlycol		X	X	
PyroglutamicAcid	Pyroglutamate	X	X	X
S-Acetamidomethylcysteine		X	X	X
SaccaricAcid			X	X
Sarcosine		X	X	
SebacicAcid		X		X
Spermidine			X	X
Taurine		X		X
Threitol		X	X	X
ThreonicAcid	Threonate	X	X	X
TMAO		X	X	X
trans-4-Hydroxy-L-Proline	Hydroxyproline	X		
UDPG			X	
Uridine			X	X
Xylitol		X	X	X

S2 Supplementary figures

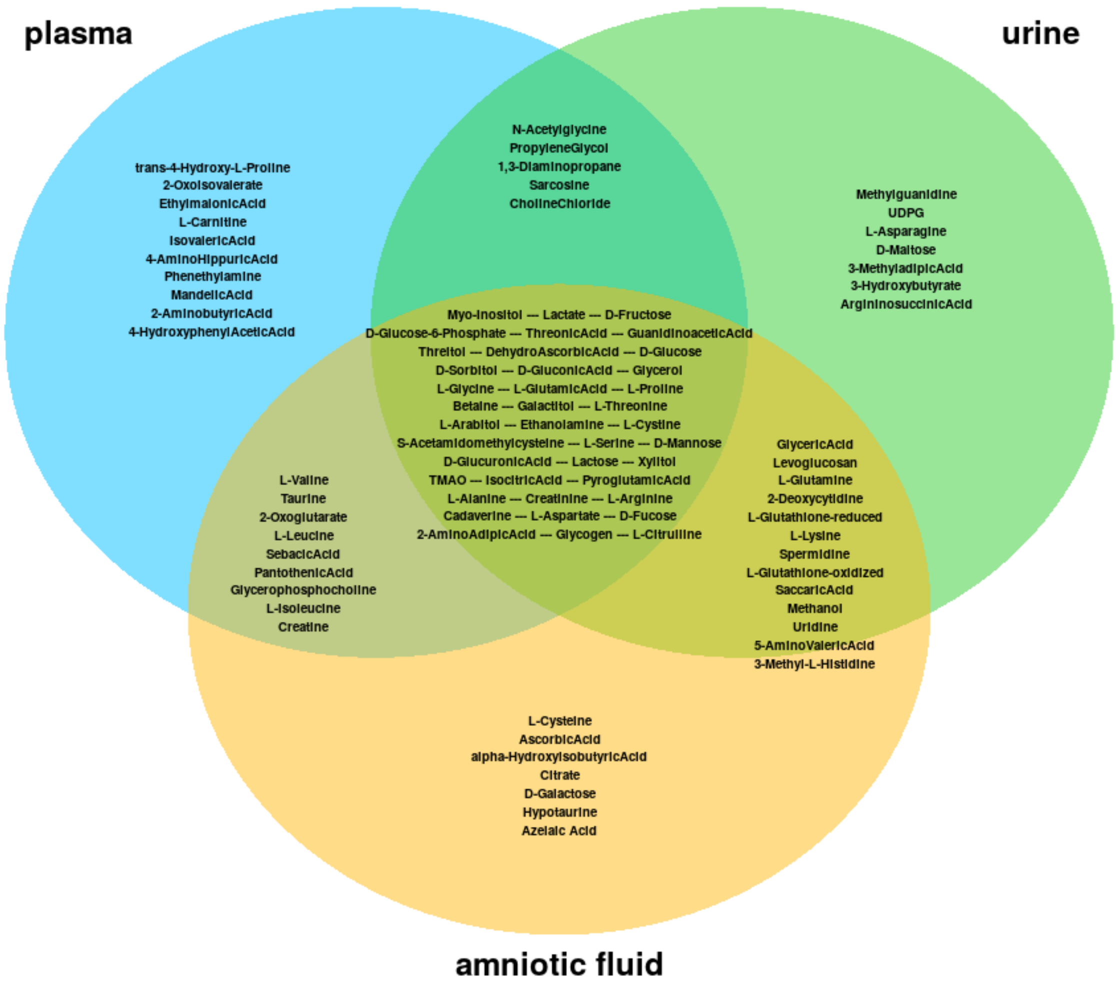

Fig. S1. Metabolites identified with **ASICS** package in each fluid.

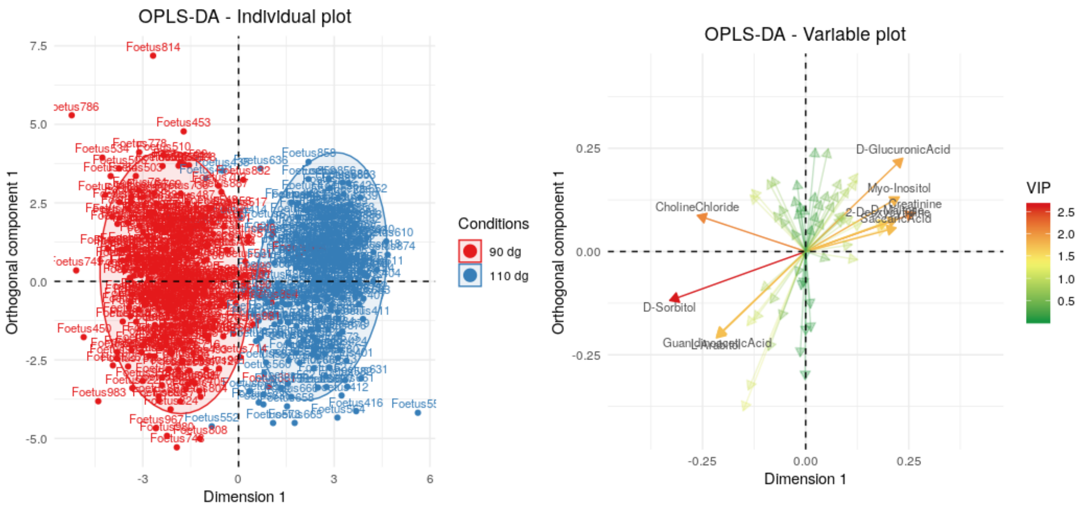

(a) OPLS-DA on urine spectra

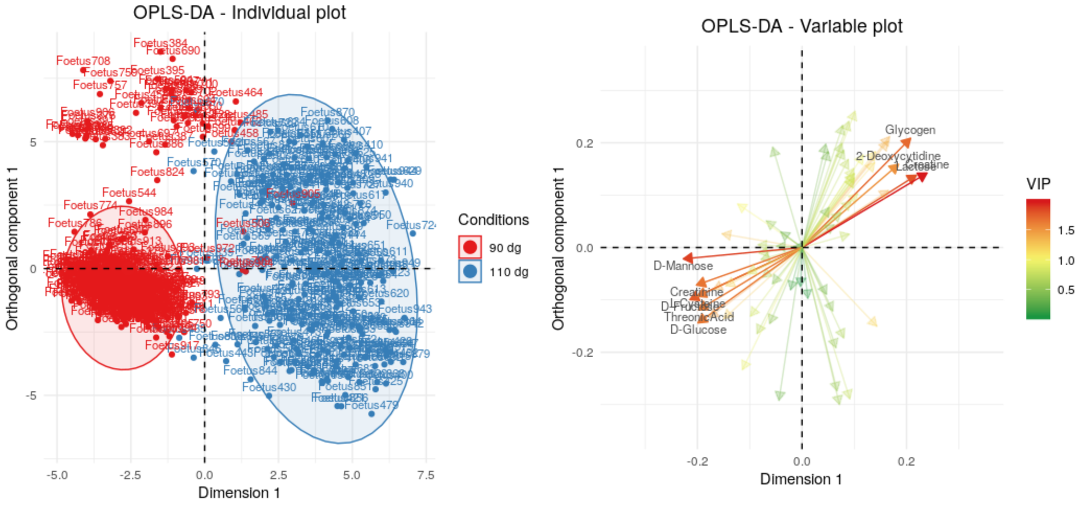

(b) OPLS-DA on amniotic fluid spectra

Fig. S2. Results of OPLS-DA.

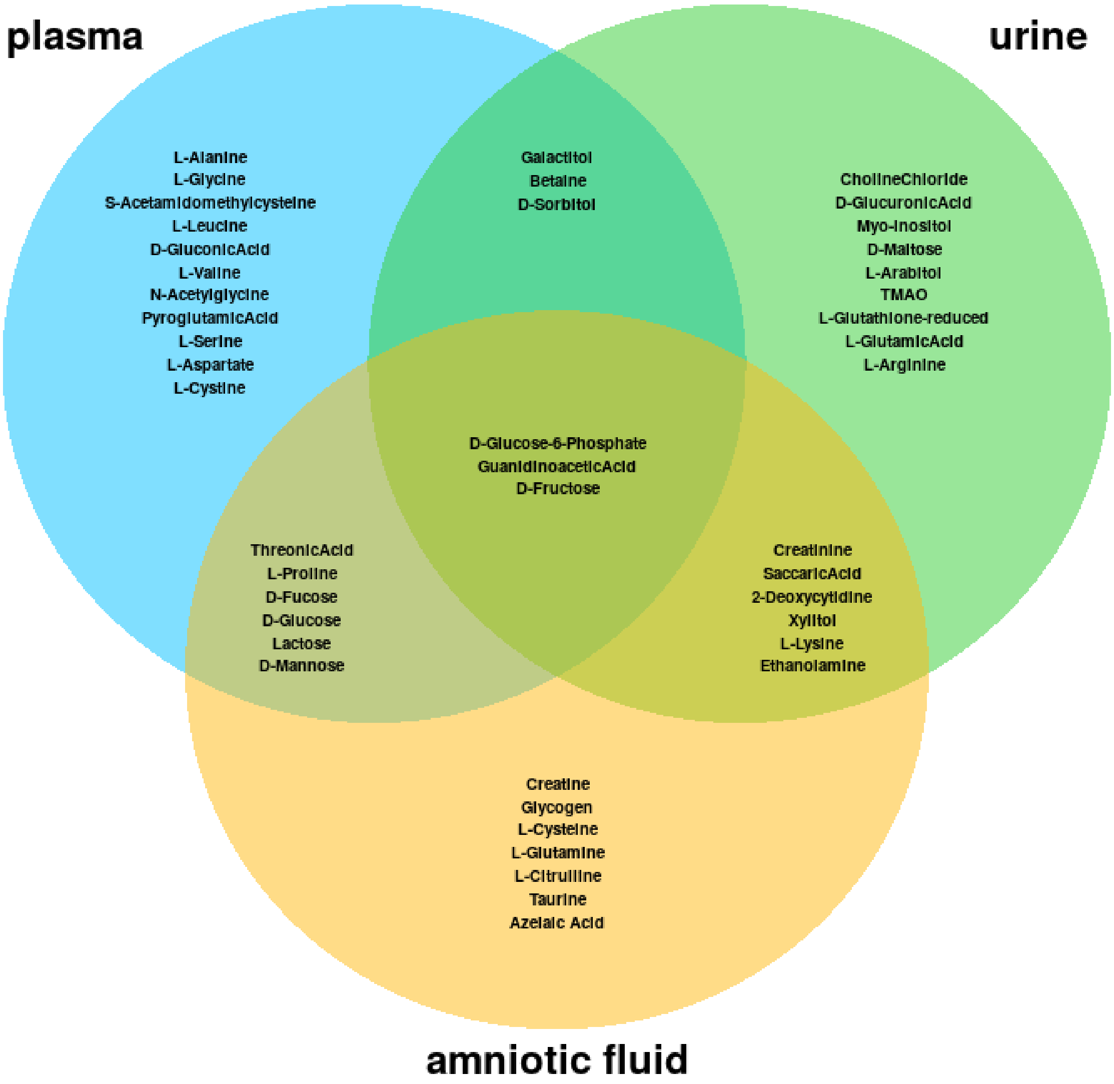

Fig. S3. Influential metabolites detected by OPLS-DA.

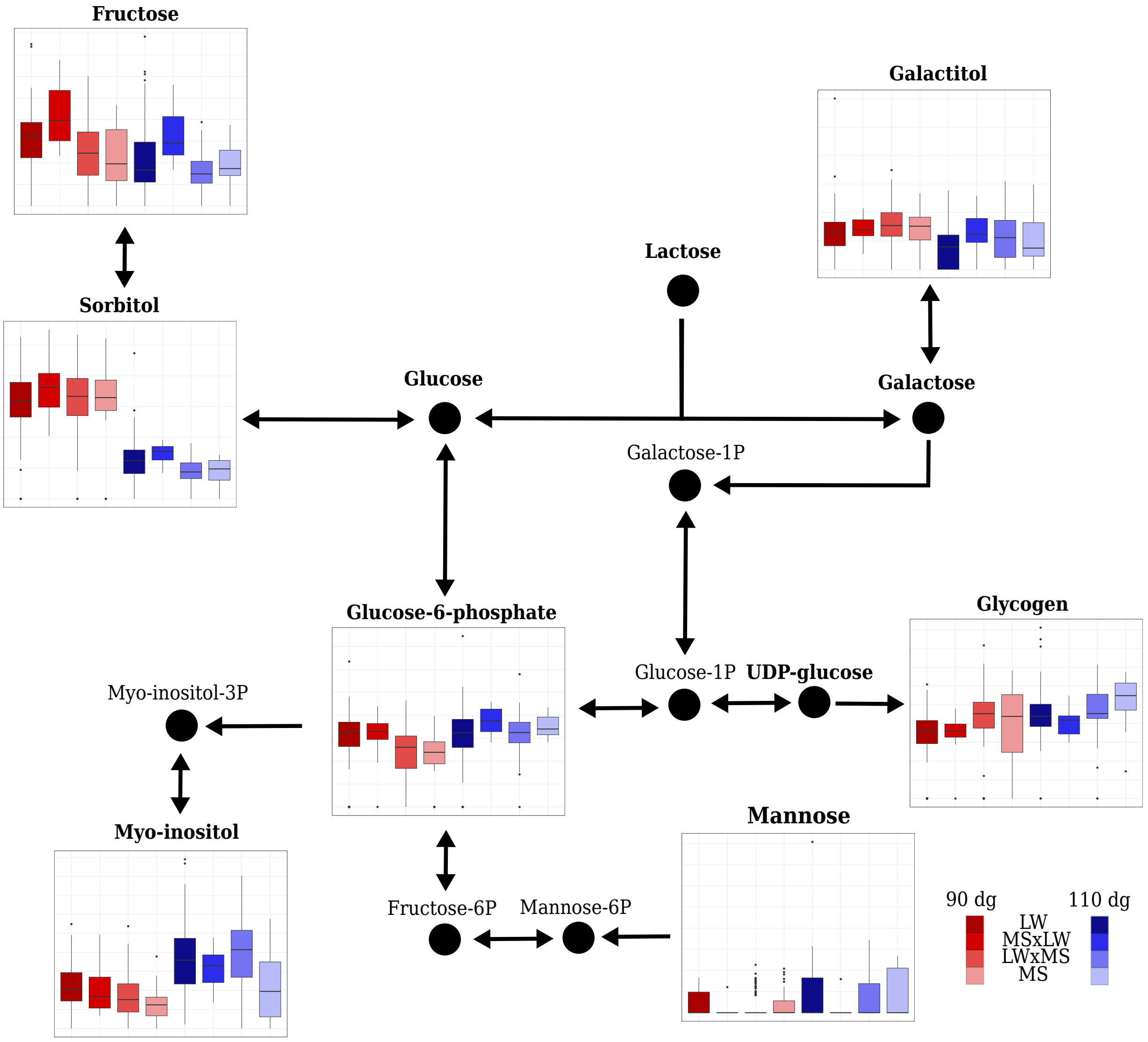

Fig. S4. Urine relative concentrations of some metabolites involved in the carbohydrate metabolism pathways ("galactose metabolism" and "starch and sucrose metabolism") according to stages of gestation (90 dg and 110 dg, in red and blue respectively) and to fetal genotypes (LW, MS×LW, LW×MS and MS, from left to right respectively). Metabolites in bold are the ones included in **ASICS** reference library. The coordinates of the y axes in boxplots can not be compared between two metabolites (relative concentrations limits of the boxplots are adapted to each metabolite).

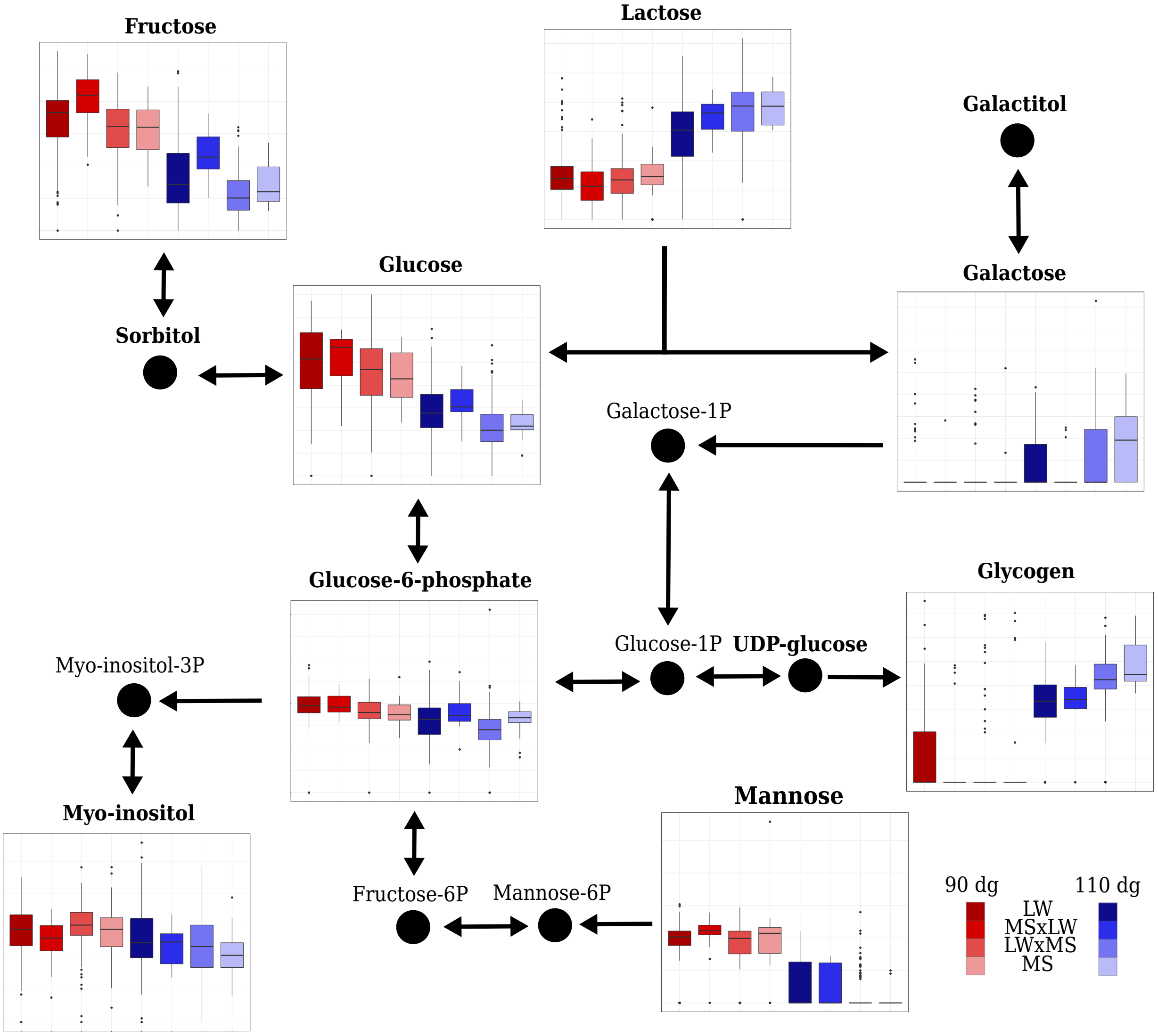

Fig. S5. Amniotic fluid relative concentrations of some metabolites involved in the carbohydrate metabolism pathways ("galactose metabolism" and "starch and sucrose metabolism") according to stages of gestation (90 dg and 110 dg, in red and blue respectively) and to fetal genotypes (LW, MS×LW, LW×MS and MS, from left to right respectively). Metabolites in bold are the ones included in **ASICS** reference library. The coordinates of the y axes in boxplots can not be compared between two metabolites (relative concentrations limits of the boxplots are adapted to each metabolite).

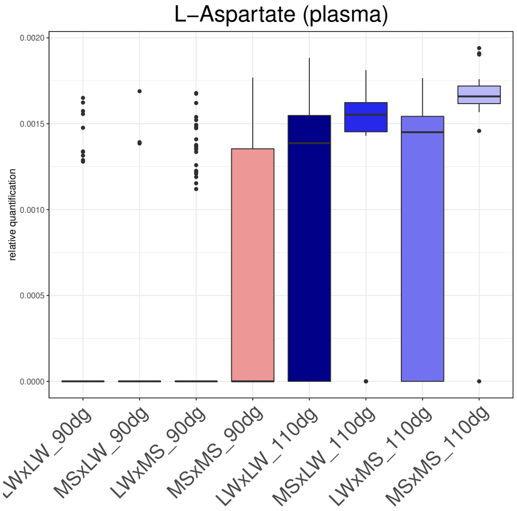

(a) Aspartate concentrations in plasma by gestational stage and genotype

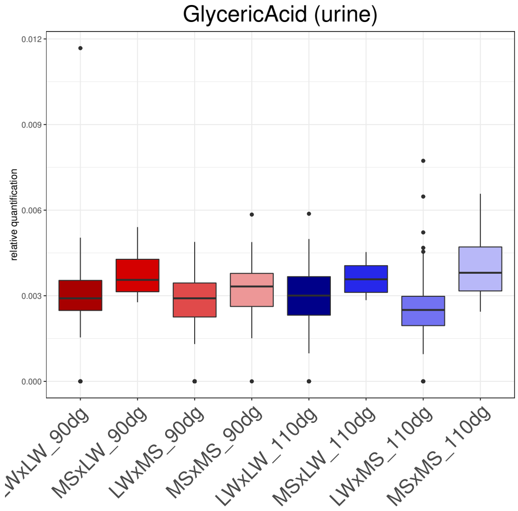

(b) Glycerate concentrations in urine by gestational stage and genotype

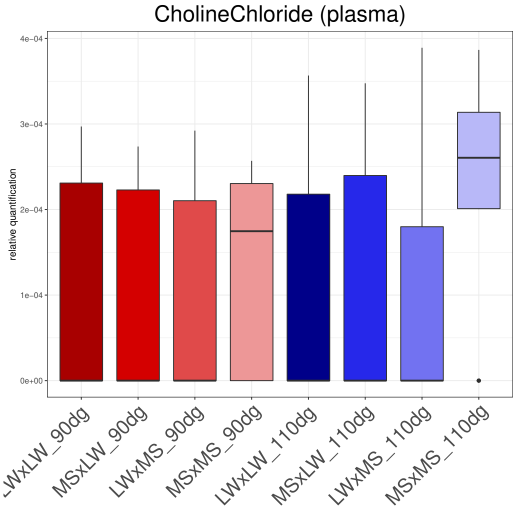

(c) Choline concentrations in plasma by gestational stage and genotype

Fig. S6. Paternal effect for aspartate in plasma and glycerate in urine and effect of the pure MS genotype for choline in plasma.

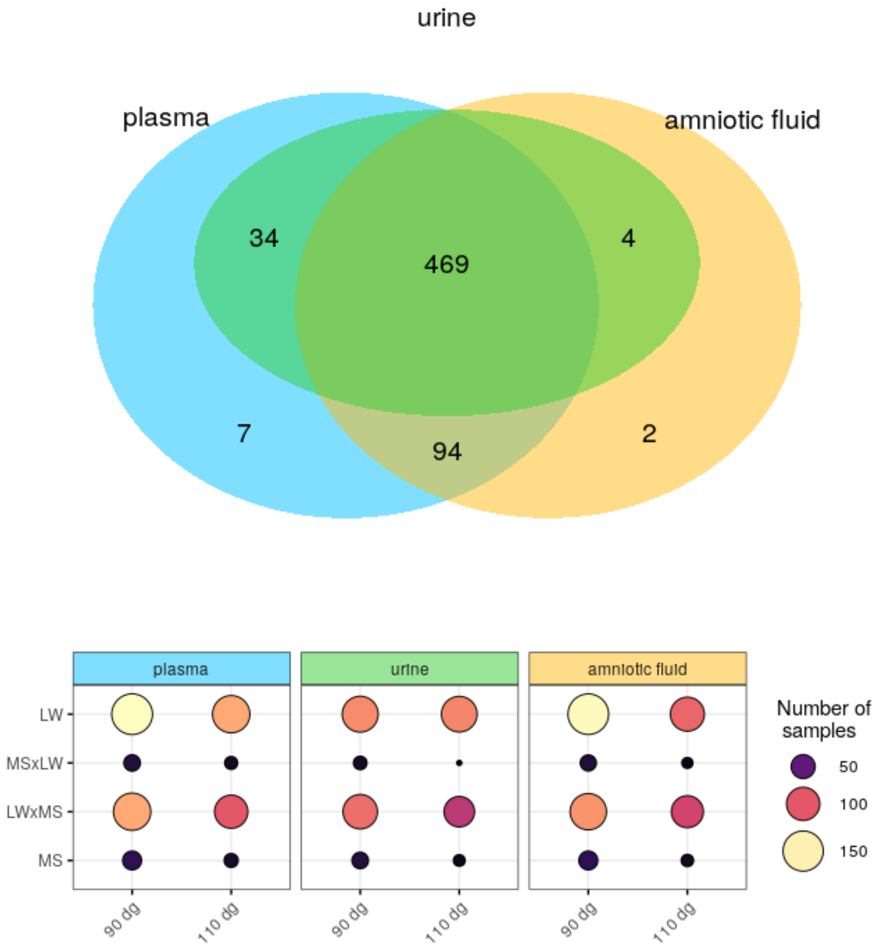

Fig. S7. Summary of the design experiment: number of samples by fluid, stage of gestation and genotype.

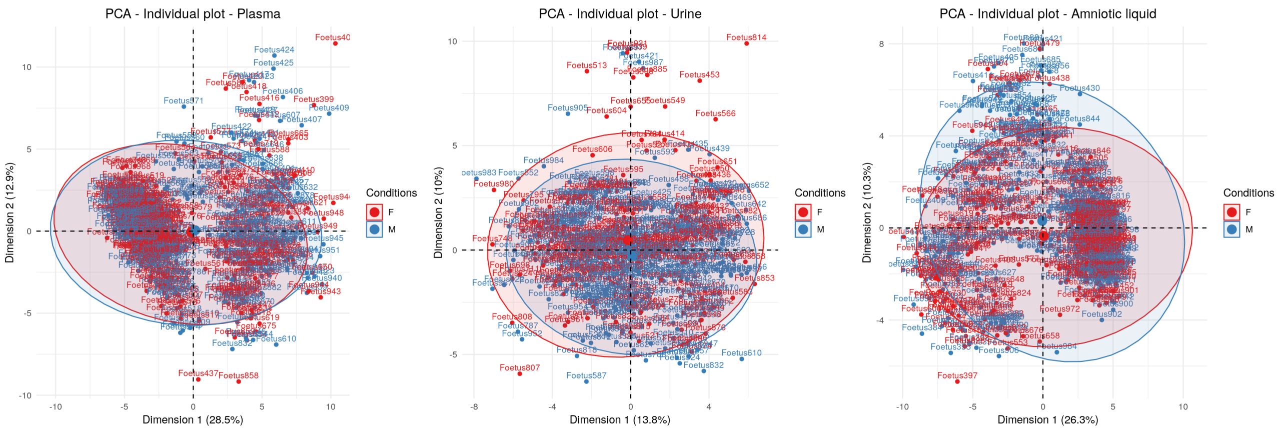

Fig. S8. PCA on quantifications of metabolites colored by gender (red: females, blue: males).

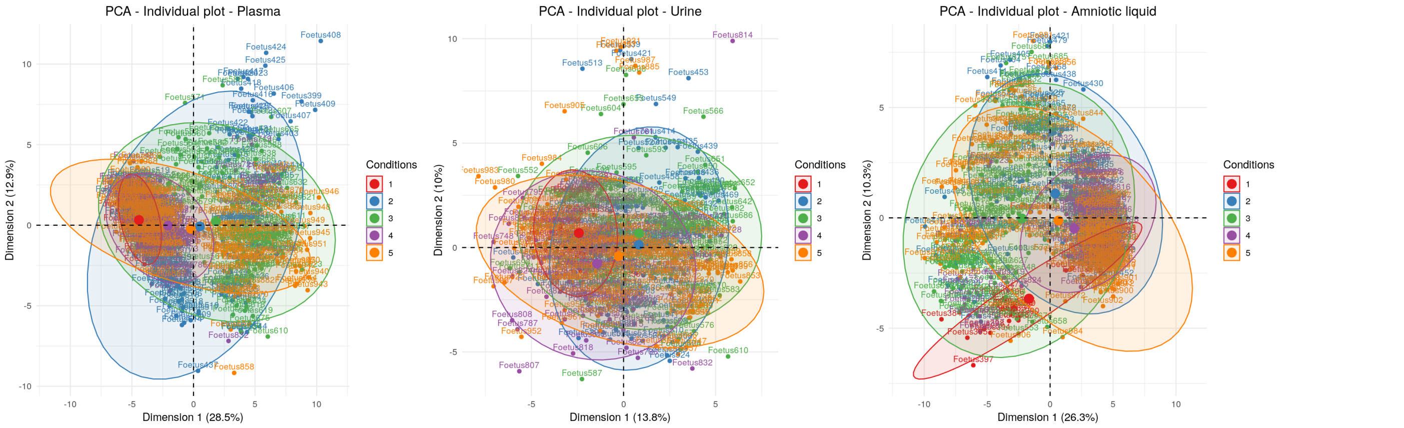

Fig. S9. PCA on quantifications of metabolites colored by batch effect.

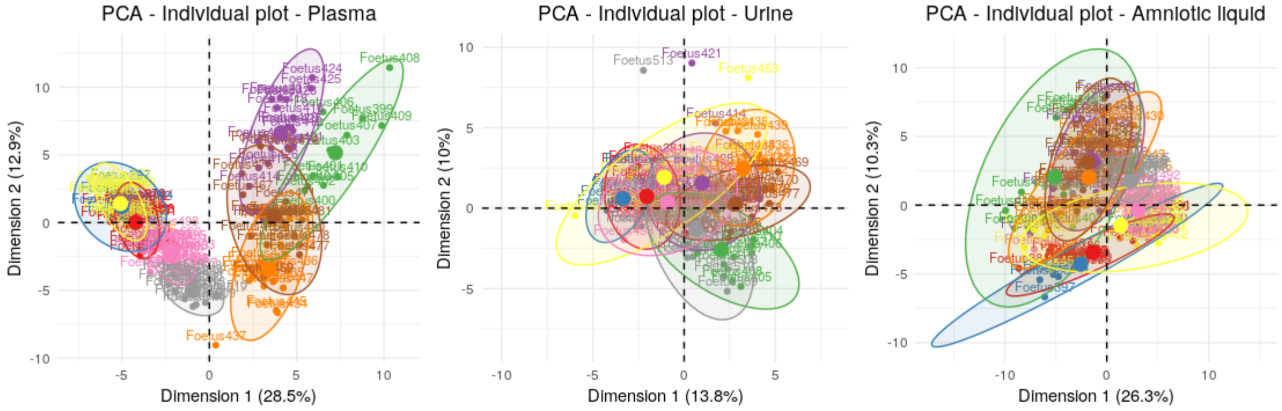

Fig. S10. PCA on quantifications of metabolites colored by sow. For the sake of clarity, only the first nine sows are represented.
